# Metabolic bone disorders and the promise of marine osteoactive compounds

**DOI:** 10.1007/s00018-023-05033-x

**Published:** 2023-12-20

**Authors:** Alessio Carletti, Paulo Jorge Gavaia, Maria Leonor Cancela, Vincent Laizé

**Affiliations:** 1 Centre of Marine Sciences (CCMAR), University of Algarve, Faro, Portugal; 2 Faculty of Medicine and Biomedical Sciences (FMCB), University of Algarve, Faro, Portugal; 3 Associação Oceano Verde (GreenCoLab), Faro, Portugal; 4 Algarve Biomedical Center (ABC), University of Algarve, Faro, Portugal; 5 Collaborative Laboratory for Sustainable and Smart Aquaculture (S2AQUAcoLAB), Olhão, Portugal; 6 Present Address: Department of Developmental Genetics, Max Planck Institute for Heart and Lung Research, Bad Nauheim, Germany

**Keywords:** Bone erosive disorders, Marine natural compounds, Marine pharmacology, Osteoanabolic compounds, Antiresorptive compounds, Osteoporosis

## Abstract

Metabolic bone disorders and associated fragility fractures are major causes of disability and mortality worldwide and place an important financial burden on the global health systems. These disorders result from an unbalance between bone anabolic and resorptive processes and are characterized by different pathophysiological mechanisms. Drugs are available to treat bone metabolic pathologies, but they are either poorly effective or associated with undesired side effects that limit their use. The molecular mechanism underlying the most common metabolic bone disorders, and the availability, efficacy, and limitations of therapeutic options currently available are discussed here. A source for the unmet need of novel drugs to treat metabolic bone disorders is marine organisms, which produce natural osteoactive compounds of high pharmaceutical potential. In this review, we have inventoried the marine osteoactive compounds (MOCs) currently identified and spotted the groups of marine organisms with potential for MOC production. Finally, we briefly examine the availability of in vivo screening and validation tools for the study of MOCs.

## Introduction

Metabolic bone disorders (MBDs) pose a significant global health challenge, with fragility fractures affecting a substantial portion of the population, notably among the elderly [1]. At the heart of fragility fractures lies the disruption of bone remodeling, an essential homeostatic process that involves the removal of old or damaged bone, followed by the deposition of new bone [2]. In the first part of this review, MBDs are described according to their impact on bone mineral density (BMD), a physiological parameter of bone health with clinical relevance [3]. Since the pathophysiological mechanisms that underlie changes in BMD are many and various, we also provide a detailed analysis of the molecular mechanisms underpinning the most common MBDs. This section also reviews the therapeutic strategies currently available for treating MBDs, assessing their efficacy and limitations, and outlines emerging pharmaceutical options. The second part of this review intends to shed some light on the potential of marine osteoactive compounds (MOCs) as natural drugs to treat MBDs. It goes through the remarkable diversity of sourced organisms and identified compounds, and gives some insights on the molecular mechanisms underlying MOC action and on drug development status. The final part of this review underscores the need for coordinated efforts between chemical characterization and the implementation of screening tools already available to explore marine organism biodiversity for bone anabolic and/or antiresorptive bioactives.

## The burden of metabolic bone disorders

In 2019, a meta-analysis of available data from 204 countries and territories reported a global incidence of fragility fractures around 2.3% of the total population and 15.4% of the elderly sub-population [1]. Bone fragility is a major concern for the global health system, causing severe disability and mortality worldwide, and placing an important financial burden on the society [4]. At the origin of fragility fractures is the dysregulation of a fundamental homeostatic process: bone remodeling. To maintain mechanical properties and architectural integrity throughout life, bone must renew senescent and damaged structures through a process requiring the concerted resorption and formation of bone mineralized matrix. An unbalance between these two processes will prompt metabolic bone disorders [2]. As different pathologies are characterized by different causing mechanisms, we will start this review with a brief description of the mineral phenotypes and molecular mechanisms underlying such disorders.

## Molecular mechanisms of metabolic bone disorders

Bone mineral density (BMD), defined as “the amount of mineral per cubic centimeter of bone tissue”, represents the gold standard in clinical practice to establish a pathological alteration of mineral content and identify patients with MBDs. Based on this clinical marker, low-BMD pathologies include *osteomalacia* [5], nutritional *rickets* [6], *osteopenia*, and *osteoporosis* [7], while high-BMD pathologies are genetic disorders united under the term *osteopetrosis* [8]. Finally, *Paget’s disease of bone* [9], *primary hyperparathyroidism* [10], and *renal osteodystrophy* [11] can be considered BMD-independent pathologies, as it has been demonstrated that they are not unequivocally diagnosed by a reduced BMD, and several manifestations of these disorders are characterized by locally elevated BMD. Although this functional classification of MBDs may be appropriate in a diagnostic setting, the therapeutic approaches adopted will mostly depend on the pathophysiological mechanisms at the origin of the disease. As such, in the following section, we have further classified bone disorders into (i) disorders affecting the mineral homeostasis through the vitamin D (VD)–parathyroid hormone (PTH) regulatory network, (ii) disorders caused by an excessive osteoclast function, and (iii) disorders induced by a defective osteoclast function.

### Disorders resulting from an altered mineral homeostasis

*Osteomalacia* and *rickets* are primarily caused by calcium or VD deficiency in adults and children, respectively [12]. Causes of these deficiencies are vast, e.g., reduced dietary intake, malabsorption in patients with gastrointestinal or liver disorders, or increased excretion induced by nephropathologies [3]. Low levels of these essential nutrients drive the mineral homeostatic system to change the source of circulating calcium from intestinal absorption to bone resorption. In this situation, PTH stimulates osteoclast differentiation by inducing an overproduction of RANKL (receptor activator of nuclear factor kappa-B ligand) and M-CSF (macrophage colony-stimulating factor) by osteoblasts, osteocytes, bone marrow stromal cells, and resident lymphocytes [13]. The persistency of this condition leads to osteopenic bones in adults and bended bones in children [14]. Osteomalacia can be rescued in adults upon VD and calcium supplementation, but bone deformities in rachitic children are often irreversible and can only be treated by surgery [15].

*Primary hyperparathyroidism* is an endocrine disorder characterized by hypercalcemia (elevated blood calcium levels) and inappropriate PTH levels, caused by benign or cancerous tumors in parathyroid glands [16]. Skeletal phenotype is characterized by loss of cortical bone, reduced BMD leading to osteopenia, and an increased risk of fracture in both vertebral and appendicular sites [17]. In the absence of suitable drugs, the only efficient cure is the surgical removal of parathyroid tissue or glands (parathyroidectomy). If surgery is not an option, a blend of calcium regulating agents, bone anabolic, and antiresorptive drugs may be used [16].

*Renal osteodystrophy* is a condition that covers skeletal disorders in patients suffering from chronic kidney disease (CKD), e.g., osteoporosis, osteomalacia, osteitis fibrosa, and adynamic bone disease [18, 19]. Initially, renal insufficiency triggers a retention of phosphorous and an accumulation of uremic toxins in blood, inducing a state of low bone metabolism known as adynamic bone disease [18]. This condition may result from the acquisition of a PTH signaling resistance by the bone tissue. The persistency of the adynamic bone condition, high level of phosphorous, and reduced circulating calcitriol (1,25-hydroxyvitamin D_3_) induces hypocalcemia and stimulates parathyroid glands, exacerbating the quantity of PTH in the serum. Patients eventually develop secondary hyperparathyroidism [19], whose histological landmarks are defined as osteitis fibrosa, which is characterized by an increased bone turnover, increased osteoblast number and activity, woven osteoid, increased osteoclast number and activity, overall increased bone resorption, low BMD, and increased fragility [18, 19].

### Disorders resulting from an excessive osteoclast activity

*Osteoporosis* (OP) and *Paget’s disease of bone* (PDB) are the most common MBDs, with a prevalence of 18.3% [20] and 0.6% [21], respectively, and both conditions result from a dysfunctional and overregulated bone resorption by osteoclasts [2]. PDB pathophysiology involves the increased formation of hyper-resorptive osteoclasts during the osteolytic and initial phase of the disease. In an attempt to recover the loss of bone mineral, the body increases bone formation, a compensatory mechanism which results in the production of an unorganized and woven bone matrix [22]. Typically, pagetic patients show a localized symptomatology (two forms—monostotic, affecting a single bone, and polyostotic, affecting more skeletal elements—exist) with a higher number of atypical osteoclasts characterized by a larger size, an increased number of nuclei, and an elevated resorptive activity. Osteoclast precursors are generally highly responsive to pro-osteoclastogenic signals such as RANKL and 1,25-(OH)_2_D_3_ and resistant to apoptotic signals [23, 24]. Clinical features of PDB include bone pain and increased serum alkaline phosphatase (ALP); microfractures and increased bone vascularization may also be observed [25], leading with time to deformations due to the weakened structure [23, 24]. Leading causes of PDB are still not fully understood, although it appears that bone formation, despite being rapid and unorganized, is in fact intrinsically normal [26]. Genetic factors associated to the disease include a plethora of mutations and variants in genes associated to osteoclast differentiation and activation, while environmental factors may include epigenetic factors, exposure to certain toxins, and infection by paramyxoviruses [27]. No cure exists for PDB, and therapeutic strategies currently available to alleviate disease symptoms focus on a set of antiresorptive drugs, mostly bisphosphonates, targeted at restoring normal levels of bone resorption. Anti-inflammatory drugs may also be implemented, as well as vitamin D and calcium supplementation, to prevent possible negative effects of the elevated bone resorption over parathyroid function, which may lead to secondary hyperparathyroidism.

Osteoporosis and osteopenia are also characterized by a dysregulated resorptive process. It is important to highlight that although we have classified osteoporosis as an “excessive osteoclast activity” disease, this disorder is characterized by a complex etiology and a variety of pathophysiological mechanisms and, in some cases, the imbalance in bone remodeling is caused by a reduced bone formation [28]. Four main pathophysiological mechanisms have been identified to be at the origin of osteoporosis, and these may overlap in some patients: postmenopausal osteoporosis, age-related osteoporosis, immobilization-induced osteoporosis, and drug-induced osteoporosis.

*Postmenopausal osteoporosis* (also known as *primary osteoporosis*) is a complex and multifactorial condition. In premenopausal women, estrogens participate in bone anabolism by inhibiting osteoblast [29] and osteocytes [30] apoptosis, thus increasing their life spam. Estrogens also prevent bone resorption by inhibiting RANKL-mediated osteoclastogenesis [31], stimulating the production of anti-osteoclastogenic cytokines by regulatory T cells [32], and inducing osteoblast-mediated osteoclast apoptosis in a paracrine manner [33]. Estrogens also excerpt a suppressive effect over thymic function, reducing the population of inflammatory T cells [34]. After the menopause, circulating estrogens are depleted as a result of reduced ovarian synthesis, and the suppressive effect they normally have over thymic function is diminished. As activated T cells produce pro-osteoclastogenic cytokines such as IL-1b and TNF-α [35], a chronically elevated bone remodeling is established at menopause, where bone resorption is not compensated by bone formation. This mechanism leads to an overall reduced BMD, increased fragility and fracture risk [36]. *Age-related osteoporosis* affects both woman and men and initiates after the peak of BMD at adolescence. Rate is similar in both genders but may be intensified in women entering menopause [37]. An hypothesis for a long time [38], there is now a growing body of evidence that support the role of an age-related increase in oxidative stress in the age-related diminution of BMD. In this scenario, reactive oxygen species (ROS) induce bone loss by stimulating osteoclast differentiation [39] and osteoblast apoptosis [40].

The term* secondary osteoporosis* is used for disorders where bone loss is a consequence of other conditions or medications [41]. It includes *immobilization-induced osteoporosis* (or disuse osteoporosis) observed in patients immobilized for a long period following illness or injuries, but also in astronauts exposed to microgravity [42]. This condition is typically characterized by cortical bone loss, while trabecular bone loss is commonly observed in other osteoporotic conditions, and is the consequence of a reduced mechanical load on bone, a physical stress mediated by the osteocytes, and altered bone remodeling [42]. It also includes *drug-induced osteoporosis*, a highly prevalent disorder associated with a prolonged drug treatment [43, 44]. Glucocorticoids are one of the best studied examples. They impair osteoblast differentiation by dysregulating the WNT/β-catenin signaling pathway [45], and also stimulate osteoblast apoptosis [46]. Indirectly, glucocorticoids affect osteoblast function by reducing the expression of insulin-like growth factor 1 (IGF-1) [47], which promotes bone formation by mediating the anabolic effects of the parathyroid hormone (PTH) [48]. Glucocorticoids can also stimulate osteoclastogenesis by reducing the production of osteoprotegerin (OPG) by osteocytes and osteoblasts [49], further favoring bone loss. Therapeutic approaches for osteoporosis comprise a set of bone anabolic and antiresorptive therapies, which are used with the main objective of preventing bone loss, increasing bone formation, and reducing the fracture risk. The advantages and disadvantages correlated to each of the major groups of pharmacological agents currently implemented will be discussed in the next section. Importantly, all therapeutics currently approved are characterized by long-term limited efficacy and side effects.

### Disorders caused by an impaired osteoclast function

These pathologies are characterized by a vast group of rare, primary monogenic disorders gathered under the name *osteopetrosis*, also known as the marble bone disease. Osteopetrosis is characterized by a defective bone resorption, increased bone mass and high BMD, and is associated with bone fragility and an increased risk of fractures, and, in some cases, with defective bone marrow, kidney, and nervous and immune systems [50]. There are two prevalent forms of osteopetrosis, which are distinguishable based on their inheritance modality. A more prevalent, milder, and typically late-onset form (arising late during childhood) known as *autosomal dominant osteopetrosis* (ADO), and a more rare, aggressive and early-onset form (arising early after birth) associated with severe phenotypes and poor prognosis, known as *autosomal recessive osteopetrosis* (ARO) [50]. ARO can be subdivided into *osteoclast-poor* and *osteoclast-rich* forms, depending on whether the mutation at the origin of the disease affects a gene linked to osteoclast differentiation or resorptive function [50]. In addition, a rare form of *X-linked osteopetrosis* (XLO) has also been described [51]. Mutations in genes that are central to osteoclast function have been associated with the etiology of osteopetrosis, in particular those involved in the acidification of bone microenvironment (*TCIRG1*, *CNCL7*), degradation of the extracellular matrix (*CTSK*), and cell differentiation (*RANK*, *RANKL*, *CSF1R*, *NEMO*, *RELA*) [52]. There are currently no pharmaceutics to efficiently treat osteopetrosis, and therapeutic approaches are only aimed at managing symptoms and relieve pain, e.g., supplementation of vitamin D and calcium in patients with hypercalcemic seizures, transfusion of red blood cells and platelets in patients with bone marrow failure, transplantation of hematopoietic stem cells in patient suffering from the most severe forms of osteopetrosis [50].

## What is on the menu? Current therapeutic strategies, their efficacy, and limitations

**Fig. 1:**
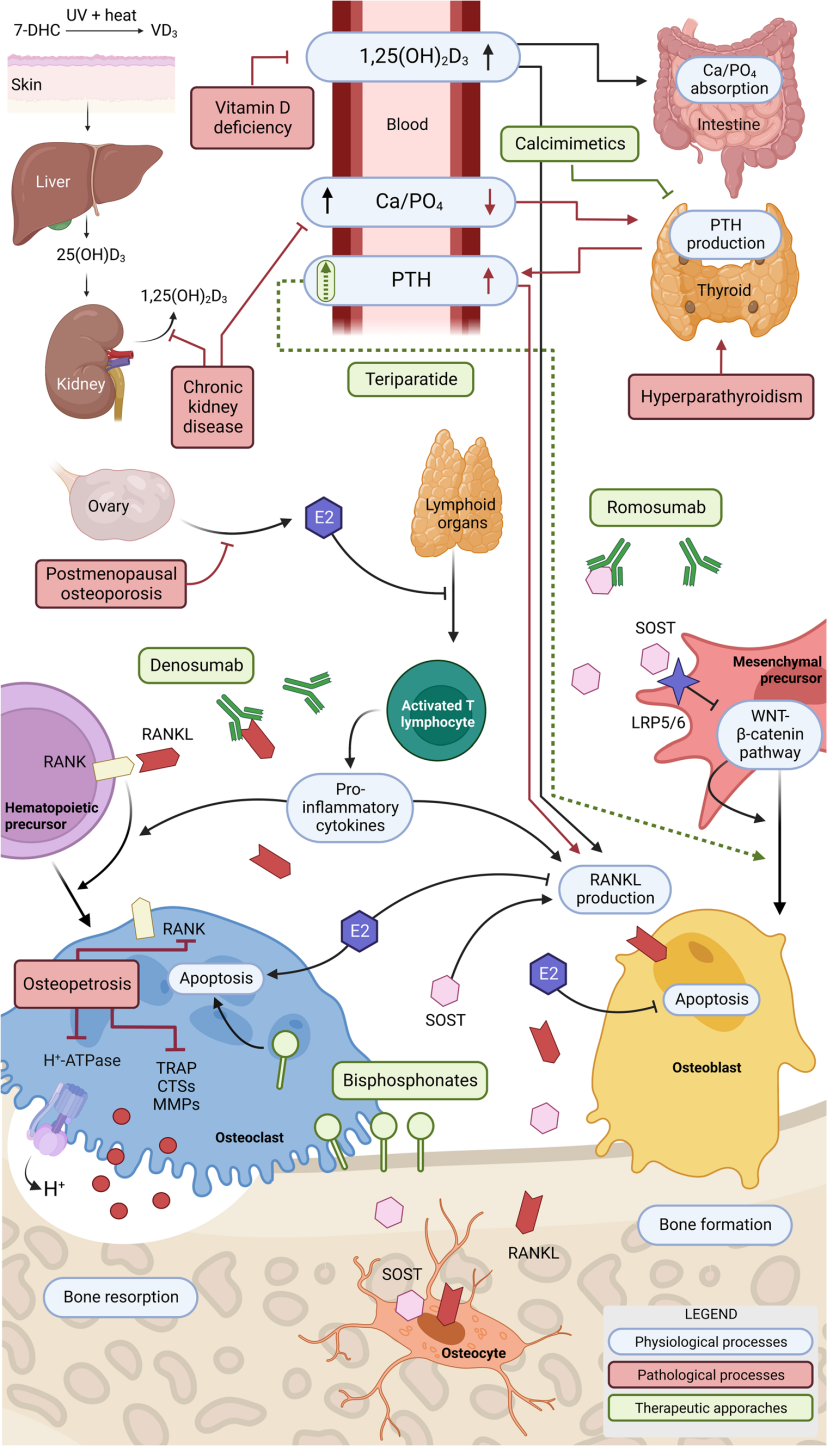
Molecular mechanisms of bone metabolic disorders (red boxes and arrows) and therapeutic treatments (green boxes and arrows) currently available. A complex network of organs, tissues, and signals intervein to control bone metabolism and a large number of emerging therapeutic targets are being described. Symbols: continuous lines with pointed arrowheads indicate process upregulation; continuous lines with blunt arrowheads indicate process downregulation; dashed lines with pointed arrowheads indicate an intermitted stimulation causing process upregulation. UV, ultraviolet radiation; Ca/PO_4_, inorganic calcium and phosphate ions; 7-DHC, 7-dehydrocholesterol; VD_3_, vitamin D_3_ (also known as cholecalciferol); 25(OH)D_3_, 25-hydroxyvitamin D_3_ (also known as calcifediol); 1,25(OH)_2_D_3_, 1,25-dihydroxyvitamin D_3_ (also known as calcitriol); PTH, parathyroid hormone; E2, estradiol; SOST, sclerostin; WNT, canonical Wnt signalling pathway; LRP5/6, low-density lipoprotein receptor-related protein 5/6; RANK, receptor activator of nuclear factor κB; RANKL, RANK ligand; H + , proton; H + -ATPase, vacuolar-type proton-ATPase; TRAP, tartrate-resistant acid phosphatase; MMPs, matrix metalloproteinase protein family members; CTSs, Cathepsins protein family members

Therapeutic solutions currently available to treat MBDs fail to meet the clinical demand. Drugs lack either efficacy or are only effective for a limited time window, or trigger long-term use-associated side effects, affecting their compatibility with the needs of patients with life-lasting chronic conditions. In the following sections, we will briefly present therapeutics currently in use, their efficacy, and limitations. Figure 1 exemplifies the main groups of bone erosive disorders, therapeutic approaches, and molecular targets.

### Vitamin D and calcium supplementation

The central roles of calcium [53] and VD [54] in bone health are long-time known. Still, there is no consensus on the dose that should be recommended to healthy individuals and patients with increased fracture risk [55, 56], nor whether benefits accompanying the supplementation of calcium and VD outweigh associated risks [57]. Calcium supplementation has little or no effect on the reduction of fracture risk in healthy individuals [58] but can reduce fracture risks and increase BMD in postmenopausal women [59, 60]. It has been associated with an increased risk of cardiovascular disease [61], although this association was refuted in a recent meta-analysis of the clinical data [62]. The source of calcium is certainly an important aspect and several studies reported that natural sources of calcium are more efficient in preventing bone loss than synthetic analogs [63]. VD supplementation, alone or in combination with calcium, has little or no effect on the reduction of fracture risk or increase of BMD in healthy individuals [64, 65] but is associated with a reduced risk of falls in elderly [66] and a reduced bone loss in postmenopausal women [67]. However, several studies highlighted that the supplementation of VD or calcium alone cannot rescue bone loss once it has already occurred [68, 69]. The combination of calcium and VD was also not associated with an increased risk of cardiovascular disease or mortality [62]. Recently, alfacalcidol [1-α-(OH)D_3_], a vitamin D_3_ analog, was found to be more effective for the treatment, rather than the prevention, of postmenopausal osteoporosis, glucocorticoid-induced osteoporosis (GIOP), and osteomalacia, when compared to cholecalciferol [70].

In relation to their application to diseases other than osteoporosis, VD and calcium supplementation represent the primary tool for the prevention and treatment of osteomalacia and nutritional rickets, and have demonstrated to be a rapid and effective therapy to restore BMD and serum biomarkers but also to relieve symptoms [71]. However, the restoration of bone density and healing of bone fractures may take time (months) and bone loss may be irreversible at some particular sites [72]. VD and calcium supplementation at low doses is also used in the treatment of primary and secondary hyperparathyroidism [73], to restore plasma levels and prevent the deficiency of both molecules in patients with abnormal PTH production or renal insufficiency. In hyperparathyroidic patients undergoing parathyroidectomy, VD and calcium supplementation is used to prevent post-surgery hypocalcemia [74]. VD supplementation also finds application in the treatment of Paget’s disease of bone, to counteract hypovitaminosis D, which appears to be frequent in pagetic patients [75], but also to prevent flu-like symptoms commonly observed in patients treated with bisphosphonates [76]. Treatment with high doses of calcitriol was tried in ARO patients to stimulate osteoclast differentiation but resulted in poor outcomes [77]. As such, its use is currently not supported by clinicians [78]. Nowadays, calcium and cholecalciferol supplementation is encouraged for osteopetrotic patients to prevent the hypocalcemic seizures that are frequently associated with this condition due to the immobility of calcium from the bone [78].

### Vitamin K supplementation

The term vitamin K (VK) collectively refers to a group of fat-soluble compounds found in animals and plants, represented by three main vitamers: phylloquinone (VK_1_), menaquinones (VK_2_), and menadione (VK_3_). The central role of vitamin K in animal physiology has been largely associated with its function as cofactor of the γ-carboxyglutamyl carboxylase (GGCX), a cytosolic enzyme which catalyzes the carboxylation of Glu into Gla residues and the functionalization of the vitamin-K-dependent proteins (VKDPs) [79], which include proteins important for bone matrix organization and mineralization such as the bone Gla protein (BGLAP or osteocalcin), matrix Gla protein (MGP), and Gla-rich protein (GRP or UCMA) [79]. Vitamin K also regulates bone metabolism in a GGCX-independent manner by binding the pregnane X receptor (PXR, SXR or NR1I2), which controls the expression of genes involved in osteoblastogenesis, osteoclastogenesis, and extracellular matrix formation and mineralization, ultimately affecting bone mechanical properties [79]. Because VK plasma levels in healthy individuals are low and detection is rather difficult, little data are available on the pathology and epidemiology of VK deficiency [79]. VK deficiency has been associated with cardiovascular disorders including neonatal bleeding [80], and vascular calcification in patients suffering from CKD [81]. In patients with end-stage CKD, VK deficiency is also associated with bone loss in the osteopenic range and increased fracture risk [82]. Other chronic disorders leading to secondary VK deficiency have also been associated with skeletal comorbidities. For instance, patients suffering from Crohn’s disease have a lower BMD associated with VK deficiency possibly due to intestinal malabsorption [83]. Despite accumulating evidence on the central role of VK in bone health, its supplementation in postmenopausal and osteoporotic patients did not significantly improve BMD and incidence of fractures [84]. Interestingly, some studies suggest that a combined treatment with VK, VD, and calcium may provide a protective effect against bone loss [85, 86].

### Supplementation of n-3 polyunsaturated fatty acids (PUFAs)

Polyunsaturated fatty acids (PUFAs) are important regulators of bone metabolism [87]. Fatty acids derivatives, such as eicosanoids and docosanoids are formed upon PUFA oxidation by cyclooxygenases, lipoxygenases, and epoxygenases, and act as anti- and pro-inflammatory molecules, respectively, regulating the equilibrium of bone remodeling [88]. For example, prostaglandin E2, a pro-inflammatory cytokine derived from arachidonic acid, can promote osteoclastogenesis and inhibit osteoblastogenesis [88]. PUFAs can also impact directly on bone cells, with n-3 PUFAs inducing proliferation of bone marrow mesenchymal stem cells while stimulating osteoblast differentiation, and n-6 PUFAs stimulating osteoclastogenesis [88]. PUFA derivates are also natural ligands of the peroxisome proliferator-activated receptor gamma (PPARγ), which is an important molecular switch that deviates the fate of mesenchymal stem cells (MSCs) from osteogenesis towards adipogenesis [88]. Multiple animal studies conducted in ovariectomized (OVX) rats and mice showed that dietary supplementation with n-3 PUFAs decreased osteoclastogenesis [89], reduced bone loss [90], and promoted chondrocyte-to-osteoblast transdifferentiaton [91].

The relative consumption of n-3 and n-6 PUFAs can also regulate the composition of bone cell membranes in fatty acids [92]. In this regard, dietary strategies that reduce n-6/n-3 ratio have been proposed for the treatment of bone erosive disorders. Two recent meta-analyses of randomized controlled trials conducted in human patients confirmed that the supplementation of n-3 PUFAs, with α-linolenic acid (ALA) being more potent than EPA and DHA, was able to slightly increase BMD, reduce resorption markers and, in the case of ALA, slightly increase bone formation markers in a short term. A stronger effect was observed in postmenopausal women [93, 94]. It is worth noting that the positive effects of PUFA supplementation reported in these studies are very low when compared with the effect of pharmaceuticals used to treat osteoporosis.

### Extracellular calcium-sensing receptor modulators

Extracellular calcium-sensing receptor (CaSR) is a central regulator of PTH secretion by the parathyroid glands in response to variations of calcium levels in the serum of higher vertebrates, and is, therefore, a key target in drug discovery for disorders characterized by the dysregulation of calcium mineral homeostasis [95]. CaSR activators, also known as calcimimetics, are molecules acting as CaSR agonists or allosteric activators*.* By binding CaSR, they inhibit PTH secretion and re-equilibrate parathyroid function in patients suffering primary, secondary, and tertiary hyperparathyroidism. Several calcimimetic drugs are used to treat hyperparathyroidism following parathyroid hyperplasia, parathyroid cancer, chronic kidney disease, and kidney transplant [95, 96]. Among those, cinacalcet has been approved for the treatment of patients with secondary and primary hyperparathyroidism that cannot or refuse to undergo parathyroidectomy. Evidences from case studies and randomized controlled trials highlighted the efficacy of cinacalcet in lowering PTH and serum calcium levels, in accordance with results in mammalian models [96]. Cinacalcet also improved bone turnover markers and bone histology but exhibited a poor ability, or none, in increasing BMD [127, 128]. Few calcimimetics are currently being evaluated in drug discovery pipelines, mainly because in vitro high-throughput technologies are missing and screening is limited to whole animal testing [95]. Calcilytics, allosteric antagonists of CaSR stimulating the secretion of PTH by the parathyroid glands, have been proposed to treat patients suffering from primary osteoporosis after several studies reported the osteoanabolic potential of transient PTH exposure [95]. Despite promising results in OVX rats [97], calcilytics did not confirm their potential in human and no reasonable advantage over PTH analogs was found. As a result, clinical trials for most of candidate calcilytics were discontinued [95, 96].

### Antiresorptive agents

Antiresorptive drugs inhibit bone resorption either by impairing osteoclast differentiation, recruitment or activity, or by promoting osteoclast apoptosis [98]. Estrogens are potent inhibitors of bone resorption and have been used in hormonal replacement therapy following menopause to increase BMD and reduce fracture risks [31]. Unfortunately, estrogen treatment was associated with an increased risk of breast and uterine cancers and cardiovascular diseases, and has progressively slipped out the list of potential treatments for postmenopausal OP [99]. Selective estrogen receptor modulators (SERMs) are drugs that can specifically modulate the activity of bone specific isoforms of the estrogen receptor; thus, they trigger the beneficial effect of estrogens over bone without increasing the risk of breast and uterine cancer [98]. Two SERMs currently approved for the treatment of postmenopausal OP, raloxifene and bazedoxifene, have demonstrated a mild positive effect on reducing fracture risk [31]. However, they have also been associated with both mild and rare but severe cardiovascular side effects [31]. Testosterone replacement therapy has proven to be effective in increasing BMD in men with osteopenia and osteoporosis [100], although several studies have associated it with an increased risk of cardiovascular diseases [101].

The peptide hormone calcitonin is a potent inhibitor of osteoclast activity [102], and both human and salmon calcitonins have been used as an antiresorptive treatment for OP, PDB, and hypercalcemia, in both injectable and nasal spray forms [103]. However, several studies associated the use of calcitonin with an increased risk of prostate cancer in men, prompting the removal of calcitonin from the list of approved therapies for osteoporosis by the European Medicine Agency (EMA) in 2012 [104]. Nowadays, calcitonin therapy is limited to pagetic patients and short treatments are recommended.

Cathepsin K (CTSK), a cysteine protease primarily involved in the degradation of bone extracellular matrix and produced in large quantities by active osteoclasts, has also been targeted by antiresorptive drugs. CTSK inhibitor odanacatib was assessed in clinical trials [105], and available data indicated a reduction of bone resorption markers and an increase of BMD in a dose-dependent manner [106]. However, positive effects quickly disappeared once the treatment was discontinued [107]. Because odanacatib was also associated with an increased risk of stroke in osteoporotic woman, all trials were discontinued [108].

*Bisphosphonates* are chemically stable analogs of inorganic pyrophosphate (PPi) with antiresorptive properties. They have been successfully used for nearly 4 decades to treat bone remodeling disorders including postmenopausal OP, age-related and immobility-induced OP, GIOP, PDB, and hyperparathyroidism [16, 98, 109, 110]. Although the implementation of bisphosphonates in clinical practice largely preceded the full understanding of their mechanism of action, an intense research effort during the last 2 decades shed some light over the molecular basis of bisphosphonate action on bone cells. Briefly, bisphosphonates bind to hydroxyapatite crystals at active sites of bone remodeling sites, then are incorporated into osteoclasts following bone resorption, where they inhibit the post-translational modification of proteins involved in cell function, ultimately leading to cell death [111]. Because of their high affinity for calcium, bisphosphonates tend to accumulate in bone, being released by osteoclasts only at active remodeling sites. Therefore, bisphosphonates are typically administered on a weekly, monthly or even yearly basis. Bisphosphonates commonly used to treat bone related disorders—alendronate, risedronate, ibandronate, and zoledronate—are able to decrease bone resorption up to 70% and reduce the incidence of vertebral and non-vertebral fractures in women with osteoporosis up to 62% and 40%, respectively [130].

*Denosumab* is a RANKL monoclonal antibody approved for the treatment of postmenopausal OP, age-related OP, and GIOP [112], but also PDB, primary and secondary hyperparathyroidism. Denosumab binds to RANKL with a high affinity, mimicking the activity of the endogenous OPG and preventing its ligation to RANK receptor at the osteoclast surface, therefore inhibiting the major signaling cascade involved in osteoclast differentiation [113]. Denosumab is a potent inhibitor of bone resorption that can reduce the incidence of vertebral, non-vertebral, and hip fracture in osteoporotic patients of 68%, 20%, and 40%, respectively, thus has an efficacy similar to that of bisphosphonates and osteoanabolic drugs [113]. As for other antiresorptive agents, patients treated with Denosumab experience a steep increase in BMD in the first 6–12 months after the beginning of the treatment, but while bisphosphonate treatment has been associated with a steady BMD after this first period, Denosumab produces a slow but continuous increase in mineral density [114]. Denosumab has also shown some efficacy in rescuing bone remodeling markers in both old and juvenile pagetic patients [115, 116], and in patients with hyperparathyroidism [117].

Bisphosphonates and Denosumab have been correlated to mild and frequent but also rare and severe side effects, raising concerns among clinicians. Among those more severe but rare, atypical femur fracture was reported in 1 patient out of 250 (frequency increases with the duration of the treatment), and osteonecrosis of the jaw was observed in 1 patient every 4000 [118]. Among those less severe but frequent upper gastrointestinal side effects, increased risk of esophageal cancer (still uncertain), musculoskeletal pain and flu-like symptoms were reported for bisphosphonates [115]. Denosumab may reduce bone turnover, a secondary effect that should be considered when treating CKD patients because of the risk of facilitating the development of adynamic bone disease [115]. Serum levels of calcium and VD must be monitored before and during Denosumab treatment due to increased susceptibility to hypocalcemia [115]. Furthermore, Denosumab treatment has been associated to increased risk of adverse effects to infections, presumably due to its immunosuppressive properties [119].

Despite their positive effect, last-generation antiresorptive drugs are characterized by a limited long-term efficacy. Indeed, although they can prevent further loss of mineral, they do not rescue the irreversible deficit in bone volume that occurs in metabolic bone disorders [114]. Several authors have proposed that the increase in BMD observed following the treatment with antiresorptive agents may only be an artefact resulting from the secondary mineralization of already-existing mineral matrix, and may not be associated with the deposition of new ECM and increase in bone volume, which are needed for structural improvement and protection against fragility fractures [114]. Furthermore, a discontinuation of antiresorptive therapy is typically associated with a re-increase in bone resorption and subsequent mineral loss [120]. As such, clinicians and researchers are currently evaluating the co-application or the sequential application of antiresorptive and osteoanabolic agents (see below).

### Osteoanabolic agents

Osteoanabolic drugs have the capacity to impact on the formation and mineralization of the extracellular matrix orchestrated by osteoblasts. It is increasingly admitted that only an osteoanabolic approach can ultimately compensate for the loss of bone volume observed in low-BMD disorders [114]. Yet, there is a surprising scarcity of bone anabolic compounds available to patients.

Among the few drugs used to restore bone mineral density, strontium ranelate was long considered the most promising osteoanabolic compound after several studies reported increased BMD and reduced fracture risk in treated patients [121]. However, its association to increased cardiovascular events and myocardial infarction in postmenopausal women led to the discontinuation of its production [122], and nowadays its use is not approved any longer by the European Medicine Agency. Two other osteoanabolic drugs are available for osteoporotic patients in Europe: teriparatide, the synthetic analog of the peptidic parathyroid hormone (PTH), and abaloparatide, the analogue of the parathyroid hormone-related peptide (PTHrP). The dualistic action of PTH on bone metabolism and the anabolic effect of an intermittent treatment with PTH—rather than the classical catabolic effect associated with the continuous exposure to PTH—is known for a long time [123]. Early studies identified osteoblastic lineage as the primary target for PTH regulation of bone homeostasis [124] and that exposure to low dosage of PTH for short periods indeed triggers the proliferation of osteoblast precursors [125]. Subsequent studies revealed that PTH stimulates osteoblast differentiation by stimulating pro-osteogenic WNT signaling pathway and inhibiting pro-adipogenic PPARγ signaling pathway in MSCs [126, 127]. PTH also inhibits apoptosis in osteoblastic cells, contributing to more cells being available for bone formation and mineralization [128]. The pro-resorptive effect of constantly elevated serum levels of PTH (e.g., during the development of hyperparathyroidism) was attributed to the stage-specific capacity of PTH to induce the expression of RANKL and inhibit OPG expression throughout osteoblast differentiation [129]. The PTH synthetic analogue teriparatide (hPTH 1–34) is composed of PTH bioactive region (amino acids 1 to 34). It is currently approved worldwide for the treatment of postmenopausal OP, age-related OP, and GIOP, and can reduce up to 80% of vertebral fracture and 50% of non-vertebral fractures in osteoporotic patients, representing one of the most effective treatment currently available [114, 130]. Teriparatide can also alleviate bone phenotypes associated with genetic disorders such as osteogenesis imperfecta [131]. Despite an excellent short-term efficacy, the long-term use of teriparatide has faced several limitations, e.g., the necessity of parenteral administration (which affect the patient’s compliance with the treatment due to side effects related to repetitive injections), and secondary effects such as decreased BMD in the radius, dizziness, leg cramps, headache and hypercalcemia [130]. Due to the dualistic effect of PTH on bone and a short-term efficacy, teriparatide will trigger an osteoanabolic effect for 12–24 months (period known as the anabolic window), then a catabolic effect characterized by increased osteoclast activity and bone resorption. Unfortunately, bone loss will occur even if treatment is discontinued [114, 132]; thus, teriparatide treatment is frequently followed by an antiresorptive therapy [114, 132].

When compared to PTH, PTHrP triggers a similar osteoanabolic action but has a milder pro-resorptive effect and a lower tendency to induce hypercalcemia. This could be related to the different affinity of PTH and PTHrP for different conformational status of the receptor PTHR1, influencing the receptor kinetic with consequence a milder stimulation of the downstream signaling cascade [130]. Based on the superior performances of PTHrP, the synthetic analog abaloparatide (PTHrP 1–34) was recently developed. It is not yet approved for the treatment of osteoporotic patients in Europe but several studies have highlighted the similar effect of teriparatide and abaloparatide in increasing BMD, and a very similar or higher effect in preventing vertebral and non-vertebral osteoporotic fractures [130]. Abaloparatide was also claimed to have a better anabolic window than teriparatide due to a lower pro-resorptive effect over time [132]. However, this claim is only supported by clinical evidence of a delayed increase in serum resorption marker C-terminal telopeptide of type 1 collagen (CTX) following Abaloparatide treatment and challenged in several studies [114]. It is worth to mention that the administration of teriparatide and abaloparatide to patients with a high risk of cancer, e.g., pagetic patients, is discouraged in the USA as it may favor the development of osteosarcoma, a warning based on studies performed in rats [133]. Yet, in 35 years of approved clinical use of teriparatide (abaloparatide was only approved in 2017), no concrete evidence of an increased incidence of osteosarcoma in humans was reported [134].

### Co-administration and sequential administration of osteoanabolic and antiresorptive drugs

Because monotherapies have shown some limitations, the efficacy of combinational therapies—i.e., the co-administration or sequential administration of antiresorptive drugs and osteoanabolic agents—has been evaluated, reviewed in [135], and results are contrasted. The co-administration of bisphosphonates and Denosumab did not clearly improve outcomes of monotreatments [98], while the combination bisphosphonate and estrogen only resulted in a slightly better BMD [135]. A recent meta-analysis of randomized controlled trials indicated that patients co-treated with teriparatide and antiresorptive agents showed an improved BMD gain and a reduced risk of fracture [136]. Sequential treatment with antiresorptive agents was only beneficial if the second treatment was done with a more potent antiresorptive; in that case, effect of the first treatment could be maintained [135]. Sequential treatments with different types of drugs have proven to be more effective. Consequently, a treatment with bisphosphonates or Denosumab following an initial treatment with bone anabolic drug could prevent bone loss commonly observed after monotherapies of osteoanabolic agents, and maintain or further increase gains in BMD [98]. However, this ideal setup has not been applied yet in clinics, where most patients are typically treated first with an antiresorptive drug, then with another antiresorptive drug or an osteoanabolic agent, whenever fracture risk is consistently high. Available evidence shows that the positive effect of teriparatide is higher in naïve patients (that never received an antiresorptive agent before) than in those receiving the treatment following an antiresorptive therapy, suggesting that the reduced rate of bone remodeling induced by antiresorptive may be blunting the remodeling-based gain in BMD triggered by osteoanabolic drugs [114]. However, the substitution of an antiresorptive therapy by an anabolic therapy appears to be overall beneficial to patients, at least regarding gain and maintenance of BMD, although the effect of this therapeutic sequence on fracture risk has yet to be evaluated [135].

### Dual-action agents

Romosumab is a human monoclonal anti-sclerostin antibody, whose use was approved in USA and EU in 2019 for osteoporotic patients presenting a high risk of fracture. Sclerostin is produced by osteocytes and serves as a master regulator of bone formation through its binding to LRP5/6 receptors and the subsequent inhibition of WNT/β-catenin canonical signaling pathway, which is paramount for osteoblast differentiation and metabolism [137]. Romosumab also increases OPG expression and consequently inhibits osteoclast differentiation [132]. Therefore, Romosumab action on sclerostin promotes bone anabolic and antiresorptive effects, which is the rationale for considering Romosumab as a dual-action drug. Clinical trials have demonstrated that Romosumab treatment induces a rapid increase in bone formation markers, an increase in BMD and an equally rapid decrease in bone remodeling markers [132]. A number of randomized controlled trials have highlighted the capacity of Romosumab to reduce the incidence of fragility fractures to an extent comparable, if not superior, to the effect of bisphosphonates and teriparatide [114, 132]. Romosumab is characterized by a short and powerful anabolic window that triggers a rapid increase in bone formation during the first months of treatment. However, after few months, Romosumab anabolic window dissipate and is substituted by a mild antiresorptive mechanism [114, 132]. As such, Romosumab treatment, similar to single-action osteoanabolic drugs, needs to be followed by the treatment with antiresorptive agents [138]. Common adverse effects of Romosumab include headache, arthralgia, and immune reactions at the injection site. An increased risk of cardiovascular events such as myocardial infarction, stroke, and cardiovascular death have been associated with Romosumab treatment [138]. Little is known about Romosumab long-term associated side effects.

### Emerging therapeutic approaches for bone disorders

Our knowledge on the molecular determinants of bone metabolism has greatly improved during the last decades, widening the spectrum of potential druggable targets to treat MBDs. Among the molecular regulators recently identified for the treatment of bone-eroding diseases, antiresorptive agents such as H^+^-ATPase suppressors and Src proto-oncogene inhibitors are promising candidates, as important factors involved in osteoclastic function [139]. Novel potential targets for osteoanabolic agents include intermediates of the WNT/β-catenin pathway such as DKK-1, GSK-3, and Sirt1, activators of the soluble guanylate cyclase (sGC), and bone morphogenetic proteins (BMPs). Hydrogen sulfide donors (H_2_S), kynurenine pathway blockers, and modulators of the osteoblast–osteoclast crosstalk (e.g., compounds impacting RANKL signaling, cell–cell interaction proteins such as Semaphorins Sema3a and Sema4D, and sphingosine-1-phosphate) are also promising candidates for the development of next-generation dual-action drugs [139].

The identification of crosstalk in cellular signaling pathways central to bone and other tissues and organs has opened the possibility to implement therapeutic strategies with a more holistic approach. Therefore, drugs targeting muscle, fat, and blood vessels are gaining momentum in the treatment of MBDs. For example, activin receptor regulators, a key component of the extracellular matrix involved in osteoclastic differentiation is being studied in animal models [139]. Myokines, factors produced by skeletal muscles, are being described for having a control over bone metabolism and might represent druggable targets for MBDs [139]. Since adipocytes and osteoblasts have a common origin, drugs able to shift the equilibrium from adipogenesis to osteogenesis in MSCs, such as TGFβ- and PPARγ-modulators, are also being evaluated [139]. Similarly, the existence of a crosstalk between endothelium and bone has shed some light on the possibility for angiogenesis regulators to be targeted by therapeutically approaches for MBDs. Among those, intermediates of the Notch signaling pathway and regulators of bone vascularization such as SLIT3 and SHN3 are being evaluated [139]. A crosstalk between gut microbiome and bone health have been identified and the capacity of probiotics and prebiotics to promote bone health has been evidenced [139, 140]. Gut microbiome has also been linked to drug efficacy [141]. Because oxidative stress and inflammation are important factors in the development of MBDs, antioxidant and anti-inflammatory compounds are increasingly being evaluated for their positive impact on bone health [142, 143]. Finally, the interaction between bone and immune system suggests that immunostimulants may also have a beneficial effect on bone [144].

Nowadays, recent advancements in the fields of molecular biotechnologies such as gene therapy, gene silencing, and regenerative medicine have led to the development of innovative biotechnological approaches for treating metabolic bone disorders. Among those, a recombinant RANKL-based vaccine has shown to be able to prevent osteoporosis in OVX mice [145]. An adenovirus-delivered microRNA-based gene silencing method was able to prevent bone loss in a mice osteoporotic model by silencing *RANK* and *CTSK* expression [146]. In addition, a gene delivery system that enhances the specific bone delivery and distribution of miRNA was also developed [147]. Stem cell transplantation technologies can also be applied to the treatment of metabolic bone disorders. In this regard, the transplantation of MSCs has shown promising results in pre-clinical studies, and clinical trials are currently being conducted in osteoporotic patients [148]. MSCs-derived extracellular vehicles (EVs) have also drawn some attention because of their osteogenic potential [149]. Hematopoietic stem cells transplantation, a well-established life-saving therapeutic option for malignant infantile osteopetrosis [150], has been recently applied to the treatment of patients suffering from the less-severe autosomal dominant form of osteopetrosis [151, 152]. A combinational strategy based on the transplantation of autologous hematopoietic stem cells where the disease-causing mutation was previously corrected through gene therapy delivered via lentivirus transformation has been adopted with success in an osteopetrotic mice model [153].

## Marine natural products as alternative players in MBD therapeutic strategies

Historically, natural products (NPs) have played a central role in the advancement of pharmacology, and they are still today the basis of many contemporary pharmaceutics. Although their use in pharmaceutical research has slowed down in the early 1990s due to technical limitations related to a poor compatibility with high-throughput screening approaches, recent biotechnological advances and the advent of the “omic” sciences have placed them back in screening pipelines for novel drugs [154, 155]. In addition, the diversity of the bioactivities found in NPs, but also their chemical novelty, and effectiveness in leading to the discovery of first-in-class medications (i.e., drugs that perform through novel and unique mechanisms of action), are features that have contributed to their leading role in drug discovery. As such, only 24.6% of all drugs approved by FDA in the last 4 decades were purely synthetic, while the remaining were either fully natural (4.6%), naturally derived (18.9%), biological (isolated from an organism/cell line or produced in a surrogate host; 18.4%), biologically produced vaccines (7.5%), natural product mimics or synthetic compounds whose bioactive portion is naturally derived (25.7%) [156]. In this new era of NP-inspired drugs, the marine environment is increasingly seen as a valuable reservoir of bioactives because of its vast yet largely unexplored biodiversity in contrast to the much more explored terrestrial environment [157].

### Animals as first-choice resources in marine pharmacology

Terrestrial plants (25%) and microorganisms (13%) are traditionally the main contributing organisms for bioactives used in disease management, in particular for bone erosive disorders [158–160]. However, animals are the primary source of compounds from the marine environment. A comprehensive review on this topic has estimated that approximately 75% of the marine compounds were isolated from invertebrates, the major phyla being Porifera (marine sponges) with 32%, and Cnidaria (e.g., corals, jellyfishes, anemones, and sea fans) with 16%. Other important groups such as Mollusca (mollusks) contributed with 5%, Echinodermata (e.g., starfish, sea urchins, and sea cucumbers) with 5%, and Chordata (e.g., tunicates and vertebrates) with 4% [161]. Despite a large untapped biodiversity, marine microorganisms contributed 22–34% of the total bioactive compounds discovered in the marine environment [161].

### Marine osteoactive compounds (MOCs)

**Fig. 2:**
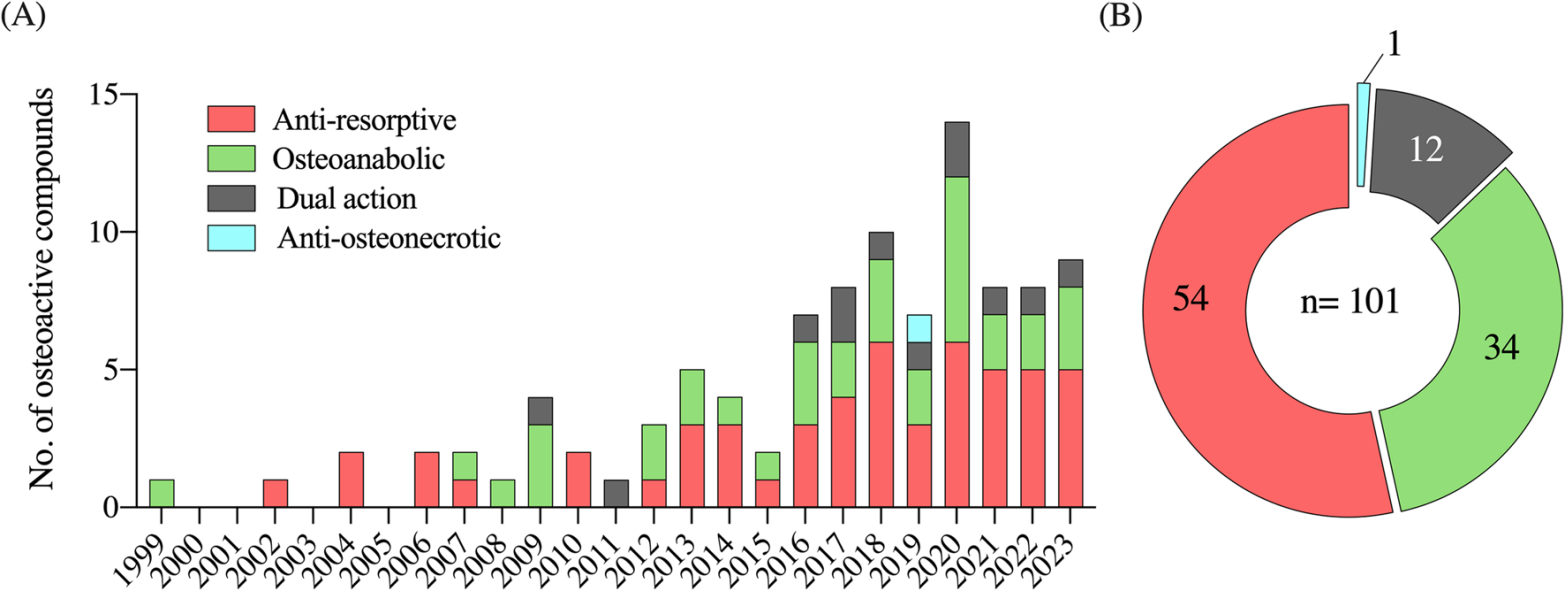
Survey of the literature available in Google Scholar regarding marine osteoactive compounds (MOCs) discovered since 1999 (**A**), and their distribution based on their mechanism of action on bone (**B**)

**Fig. 3:**
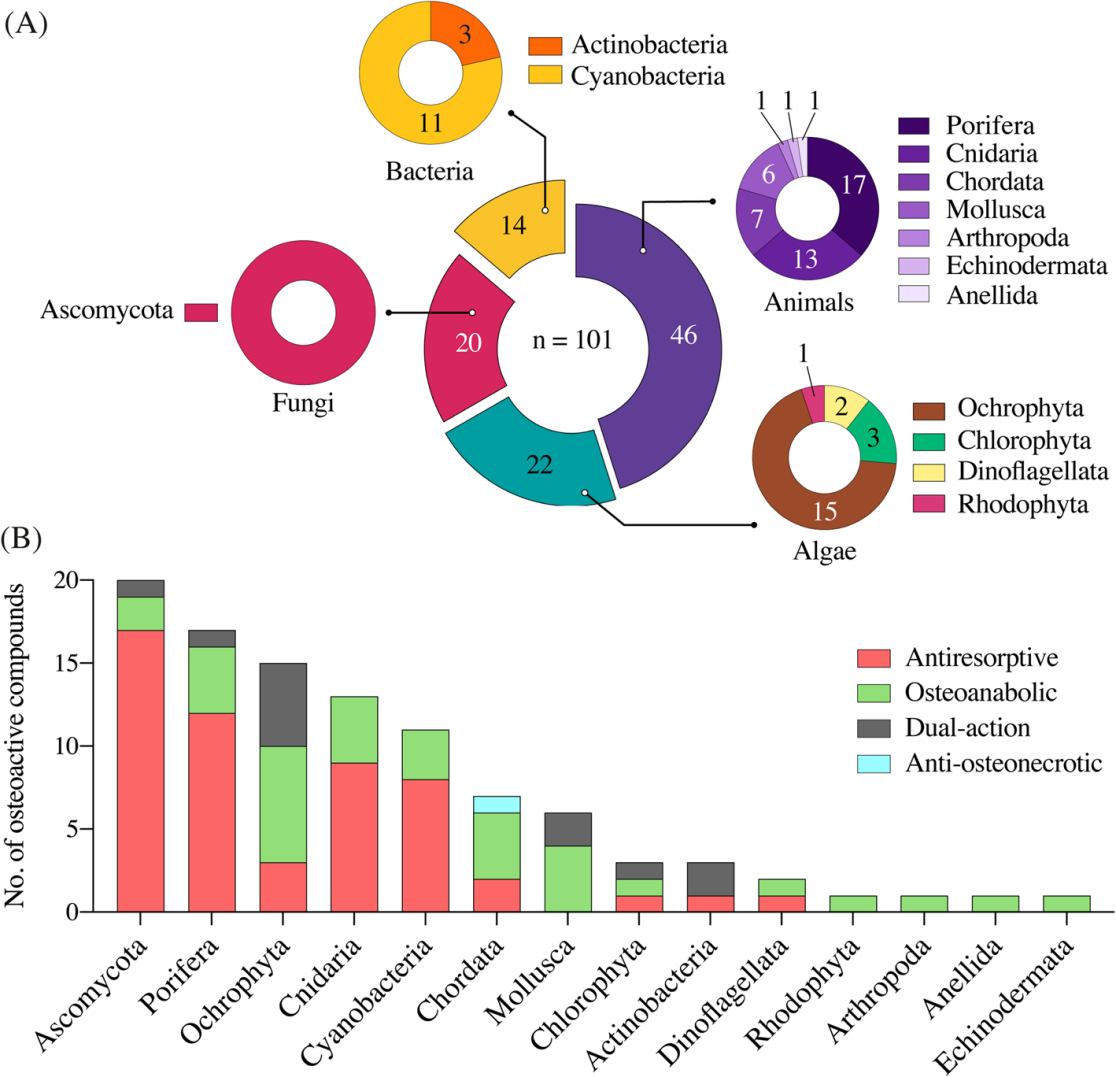
Taxonomic distribution of the species that produced the marine osteoactive compounds (MOCs) reported in the literature from 1999 to 2023 (**A**) and the number and type of MOCs described by Phylum (**B**)

Compounds isolated from marine organisms hold a great potential for the treatment of MBDs [162]. Still, limited research effort has been put on the discovery of marine compounds with osteoactive properties. This section will review the literature data on the isolation of marine osteoactive compounds from 1999 to 2023. Note that only compounds with pharmacological applications will be presented here; marine-derived biomaterials with applications in bone regeneration, fracture healing, and tissue engineering will be overlooked since it has already been reviewed [163–166]. Our survey identified a total of 101 marine osteoactive compounds (Fig. 2B), of which 54 (53.5%) are antiresorptive, 34 (33.7%) are osteoanabolic, 12 (11.9%) have a dual-action, and 1 (0.9%) is anti-osteonecrotic (Table 1). Our survey also revealed an overall scarcity of studies, with only 90 papers published between 1999 and 2023 about the isolation of new MOCs. However, the last 2 decades have seen a steadily increase in these studies (Fig. 2A), which is in agreement with the overall increment of all-type marine bioactives reported previously [157]. As such, a significant increase in the research effort aiming at the discovery of osteoactive compounds from marine organisms is anticipated in the upcoming years. The taxonomic distribution of the organisms contributing to MOCs is shown in Fig. 3. Animals (46 compounds, mostly from invertebrates) are the largest contributors (Fig. 3A), followed by algae (22, mostly from large pluricellular brown algae), fungi (20, all from ascomycetes), and bacteria (14, mostly from cyanobacteria). The distribution of MOCs at Phylum level (Fig. 3B) revealed that fungi (Ascomycota) and sponges (Porifera) provided the highest number of MOCs (20% and 17%, respectively), followed by brown macroalgae (Ochrophyta, 14.9%), corals (Cnidaria, 12.9%), cyanobacteria (10.1%), Chordata (6.9%) and Mollusca (5.9%). Dinoflagellates (Dinoflagellata), green- and red algae (Chlorophyta and Rhodophyta), crustaceans (Arthropoda) and worms (Anellida) collectively accounted for the remaining MOCs (4%). This data, although limited to a reduced set of compounds, validates the suitability of marine organisms as sources of natural bioactives for marine pharmacology.

**Table 1:** Marine osteoactive compounds described in the period 1999–2023 that could be used to treat metabolic bone disorders

Effect	Compound	Molecular mechanism	Source group	Model for screening^a^	Tested on a disease model?	Lower bioactive dose^b^	Drug discovery stage	Reference
Anti-resorptive	Salinosporamide A	Inhibition of RANKL-induced osteoclastogenesis	Actinobacteria	RAW 264.7	Not tested	50 nM	Pre-clinical	[167]
	Biselyngbyaside	Inhibition of RANKL-induced osteoclastogenesis via c-Fos and NFATC1 inhibition. Reduction of pit formation. Stimulation of osteoclast apoptosis via Caspase-3 and nuclear condensation induction	Cyanobacteria	RAW 264.7	Not tested	3 nM	Pre-clinical	[168]
	Irijimaside A-E	Inhibition of RANKL-induced osteoclastogenesis. Reduced TRAP activity	Cyanobacteria	RAW 264.7	Not tested	10 μM	Pre-clinical	[169]
	Bromo-honaucin A	Inhibition of RANKL-induced osteoclastogenesis through Akt inhibition and ERK activation. Downregulation of osteoclast markers (*Ctsk*, *Mmp9, Dcstamp*). Suppression of pit formation	Cyanobacteria	RAW 264.7	Not tested	0.1 μg/mL	Pre-clinical	[170]
	Kalkitoxin	Inhibition of RANKL-induced osteoclastogenesis. Reduced pit area and actin ring formation. Downregulation of osteoblast markers (*Mmp9*, *Acp5, Dcstamp,* CTSK, NFATC1, FOS). Inhibition of MAPK and AKT pathways. Prevention of bone loss, restoration of BMD and bone microarchitecture	Cyanobacteria	mDM-BM	Mouse model of LPS-induced bone loss	1 mg/kg/day (mouse)	Pre-clinical	[171]
	Symbioimine	Inhibition of RANKL-induced osteoclastogenesis	Dinophyceae	RAW 264.7	Not tested	44 μg/mL	Pre-clinical	[172]
	Sulfated glucurono-rhamnoxylan polysaccharide	Inhibition of RANKL-induced osteoclastogenesis. Reduced TRAP activity and actin ring formation. Downregulation of MMP9, CTSK, TRAF6, GSN, CA II, ITGB3. Suppressed activation of PTK2, CBL. Increased BMD and OPG/RANKL ratio in OVX mice	Ulvophyceae	RAW 264.7	OVX mouse	400 mg/kg/day (mouse)	Pre-clinical	[173]
	Fucoxanthin	Inhibition of RANKL-induced osteoclastogenesis. Stimulation of osteoclast apoptosis via Caspase-3 induction. Modulation of MAPK and Nrf2 pathways	Phaeophyceae	RAW 264.7; MC3T3-E1	OVX rat	20 mg/kg/day (rat)	Pre-clinical	[174–176]
	Sargachromanol G	Inhibition of RANKL-induced osteoclastogenesis. Downregulation of osteoclast markers (*Acp5*, *Ctsk*, *Mmp9*, *Calcr*). Inhibition of RANKL-mediated IκBa degradation and MAPK pathway activation	Phaeophyceae	RAW 264.7	Not tested	10 μM	Pre-clinical	[177]
	Glucuronomannan oligomers (Gs)	Inhibition of RANKL-induced osteoclastogenesis through the upregulation of IRF-8	Phaeophyceae	RAW 264.7	Not tested	10 μg/mL	Pre-clinical	[178]
	Mycoepoxydiene	Inhibition of RANKL-induced osteoclastogenesis. Inhibition of *Nfatc1* expression through the suppression of TAK1 phosphorylation. Inhibition of NF-κB and ERK1/2 pathways	Sordariomycetes	Murine primary bone marrow cells	OVX mouse	4 mg/kg/day (mouse)	Pre-clinical	[179]
	Stachybotrysin	Inhibition of RANKL-induced osteoclastogenesis. Inhibition of ERK, JNK, and p38 phosphorylation	Sordariomycetes	mDM-BM	Not tested	5 μg/mL	Pre-clinical	[180]
	Macrolides 1,5,9	Inhibition of RANKL-induced osteoclastogenesis	Sordariomycetes	mDM-BM	Not tested	1 μM	Pre-clinical	[181]
	Chlovalicin	Inhibition of RANKL-induced osteoclastogenesis	Sordariomycetes	mDM-BM	Not tested	0.1 μM	Pre-clinical	[182]
	Griseofulvin	Inhibition of RANKL-induced osteoclastogenesis through NF-κB pathway inhibition	Sordariomycetes	mDM-BM	Not tested	10 μM	Pre-clinical	[183]
	Insulicolide A	Inhibition of RANKL-induced osteoclastogenesis in vitro and bone resorption in vivo*.* Inhibition of IκBa phosphorylation, and NF-κB, p65, and RelB nuclear translocation. Reduction of *Dcstamp* expression. Inhibition of RANKL-induced up-regulation of NFATc1, DC-STAMP	Eurotiomycetes	RAW 264.7	Mouse model of LPS-induced osteolysis	5 mg/kg/day (mouse)	Pre-clinical	[184]
	6-epi-Notoamide T	Inhibition of RANKL-induced osteoclastogenesis. Downregulation of osteoclasts markers (*Nfatc1*, *Acp5*, *Ctsk*, *Atp6v0d2*, *Dcstamp*, *Ocstamp*)	Eurotiomycetes	RAW 264.7	Not tested	5 μM	Pre-clinical	[185]
	Austalide V-X	Inhibition of RANKL-induced osteoclastogenesis	Eurotiomycetes	mDM-BM	Not tested	3 μM	Pre-clinical	[186]
	Chlorinated polyketide 2,7	Inhibition of LPS-induced NF-κB activation and RANKL-induced osteoclastogenesis in macrophages	Eurotiomycetes	RAW 264.7; mDM-BM	Not tested	20 μM	Pre-clinical	[187]
	Taichunins G,K,N	Inhibition of RANKL-induced osteoclastogenesis	Eurotiomycetes	RAW 264.7	Not tested	5 μM	Pre-clinical	[188]
	Mactanamide	Inhibition of RANKL-induced osteoclastogenesis	Eurotiomycetes	mDM-BM	Not tested	10 μg/mL	Pre-clinical	[189]
	Steckwaic acid F	Inhibition of RANKL-induced osteoclastogenesis via NF-κB inhibition	Eurotiomycetes	mDM-BM	Not tested	10 μg/mL	Pre-clinical	[190]
	Agelasine D	Inhibition of RANKL-induced osteoclastogenesis. Downregulation of osteoclast markers (*Fos*, *Nfatc1*, *Acp5*, *Ctsk*, *Mmp9, Dcstamp*, *Ocstamp*). Inhibition of pre-osteoclast fusion, ERK phosphorylation and NF-κB activation	Porifera	mDM-BM	Not tested	3 μM	Pre-clinical	[191]
	Placotylene A	Inhibition of RANKL-induced osteoclastogenesis through the inhibition of NFATc1 transcription and translation	Porifera	mDM-BM	Not tested	3 μM	Pre-clinical	[192]
	Halenaquinone	Inhibition of RANKL-induced osteoclastogenesis through the suppression of IκBa degradation and Akt phosphorylation	Porifera	RAW 264.7	Not tested	20 μM	Pre-clinical	[193]
	Haploscleridamine	Inhibition of cathepsin K activity	Porifera	Cathepsin K inhibitor assay	Not tested	–	Pre-clinical	[194]
	Ceylonamide A,B	Inhibition of RANKL-induced osteoclastogenesis	Porifera	RAW 264.7	Not tested	10 μM	Pre-clinical	[195]
	Ceylonin A	Inhibition of RANKL-induced osteoclastogenesis	Porifera	RAW 264.7	Not tested	50 μM	Pre-clinical	[196]
	Aaptamines	Inhibition of RANKL-induced osteoclastogenesis	Porifera	RAW 264.7	Not tested	5 μM	Pre-clinical	[197]
	Neviotine A,D	Inhibition of RANKL-induced osteoclastogenesis	Porifera	RAW 264.7	Not tested	20 μM	Pre-clinical	[198]
	Amakusamine	Inhibition of RANKL-induced osteoclastogenesis	Porifera	RAW 264.7	Not tested	20 μM	Pre-clinical	[199]
	Aaptocarbamates A-G	Inhibition of RANKL-induced osteoclastogenesis	Porifera	RAW 264.7	Not tested	20 μM	Pre-clinical	[200]
	11-Episinulariolide acetate	Anti-inflammatory activity in LPS-stimulated macrophages through the inhibition of iNOS and COX2 expression. Attenuation of phenotype histological and anatomical landmarks. suppression of *Ctsk, Mmp-9, Alpl, Tnf* expression in murine models of adjuvant-induced arthritis	Cnidaria	RAW 264.7	Rat model of rheumatoid arthritis	9 mg/kg/day (rat)	Pre-clinical	[201]
	Junceellolide D	Inhibition of RANKL-induced osteoclastogenesis through the increased stability and nuclear translocation of NRF2. Inhibition of RANKL-induced generation of ROS and activation of NF-κB and MAPK pathways	Cnidaria	mDM-BM	Not tested	–	Pre-clinical	[202]
	Excavatolide B	Inhibition of LPS-induced osteoclastogenesis. Downregulation of osteoclast markers (TRAP, *Ctsk*, *Mmp9*). Rescue of clinical and histopathological features of adjuvant-induced and collagen-induced arthritis. Inhibition of osteoclastogenesis through the suppression of NFATc1 signalling in cartilage and synovial tissues	Cnidaria	RAW 264.7	Rat models of adjuvant- and collagen-induced arthritis	2.5 mg/kg/day (rat)	Pre-clinical	[203]
	Orsaldechlorin A,B	Inhibition of LPS-induced NF-κB activation. Inhibition of RANKL-induced osteoclastogenesis	Cnidaria	RAW 264.7	Not tested	15 μM	Pre-clinical	[204]
	Secosteroids 2,11,12	Inhibition of RANKL-induced osteoclastogenesis	Cnidaria	mDM-BM	Not tested	0.5 μM	Pre-clinical	[205]
	Briarane-type diterpenoids	Inhibition of RANKL-induced osteoclastogenesis through the upregulation of Nrf2 pathway. Rescued bone loss in a GIOP zebrafish		mDM-BM	Zebrafish GIOP model	–	Pre-clinical	[206]
	Iejimalide A,B	Suppression of RANKL-induced osteoclastogenesis through the inhibition of V-ATPase	Chordata	mDM-BM	Not tested	0.01 μM	Pre-clinical	[207]
Osteo-anabolic	Neotricitrinols A-C	Stimulation of osteoblastogenesis. Inhibition of adipogenesis	Eurotiomycetes (Ascomycota)	mMSC-BM	Not tested	–	Pre-clinical	[208]
	Penicopeptide A (PPA)	Stimulation of osteoblastogenesis in vitro and rescue of bone loss in vivo through the activation of AKT/GSK-3β/β-catenin pathway	Eurotiomycetes (Ascomycota)	mMSC-BM	OVX mouse	10 mg/kg/day (mouse)	Pre-clinical	[209]
	Largazole	Inhibition of histone deacetylases. Downregulation of *Bmp-2, 4, 6, 7,* and* 9*. Upregulation of *Runx2*, *Alp*, *Opn* in C2C12. Osteogenic properties during mouse calvaria regeneration and rabbit calvaria fracture healing	Cyanobacteria	C2C12; Mouse bone formation assay; Rabbit bone fracture healing assay	Not tested	2.5 μM	Pre-clinical	[210]
	Majusculamide A,B	Stimulation of osteoblastogenesis and ALP activity	Cyanobacteria	MC3T3-E1	Not tested	7.5 μM	Pre-clinical	[211]
	Amphirionin-5	Stimulation of osteoblast proliferation	Dinophyceae	MC3T3-E1	Not tested	0.001 ng/mL	Pre-clinical	[212]
	Floridoside	Stimulation of osteoblastogenesis and formation of mineralized nodules. Upregulation of *Bmp-2*, *Runx2*, *Sp7*, *Col1a1*, *Alpl*, *Bglap*, *Spp1*; Stimulation of COL1A1 production and ALP activity	Florideophyceae	D1	Not tested	0.1 μM	Pre-clinical	[213]
	*Dunaliella salina*-derived peptide P32 (ALVFQAQH)	Improved craniofacial skeleton development and mineralization in GIOP zebrafish. Upregulation of osteoblast markers (*runx2*, *alpl*, *bglap*) and downregulation of antioxidant response markers (*cat*, *sod1*). Improved BMD and bone microarchitecture in OVX rats	Chlorophyceae	Zebrafish GIOP model	OVX rat	100 mg/kg/day (rat)	Pre-clinical	[214]
	*Nannochloropsis oculata*-derived tetrameric peptide	Stimulation of osteoblastogenesis. Upregulation of osteoblast markers (ALP activity, *BGLAP COL1A1*, *BMP-2*, *BMP4*). Increased bone mineralization and phosphorylation of MAPK and SMAD pathway in both MG-63 and D1	Eustigmatophyceae	MG-63; D1	Not tested	37.5 μM	Pre-clinical	[215]
	Phlorotannins 1,2	Stimulation of osteoblastogenesis. Increased ALP activity, ECM mineralization, total protein and collagen synthesis	Phaeophyceae	MG-63	Not tested	0.1 μM	Pre-clinical	[216]
	Dioxinodehydroeckol	Stimulation of osteoblast proliferation and differentiation, and ECM mineralization. Upregulation of osteoblast markers (*Alp, Bmp2, Col1a1, Bglap*). Stimulation of Smad, ERK, Runx2 pathways	Phaeophyceae	MC3T3-E1	Not tested	20 μM	Pre-clinical	[217]
	Sargahydroquinoic and sargaquinoic acids	Stimulation of osteoblast proliferation and differentiation, and upregulation of osteoblast markers. Inhibition of adipogenic differentiation, lipid accumulation and downregulation of adipocyte markers in 3T3-L1 cells	Phaeophyceae	MC3T3-E1; 3T3-L1	Not tested	50 μg/mL	Pre-clinical	[218]
	Phlorofucofuroeckol A	Stimulation of osteoblast proliferation and differentiation, and ECM mineralization. Upregulation of osteoblast markers (*ALP, BMP-2, BGLAP*) through the stimulation of Wnt/β-catenin pathway	Phaeophyceae	hMSC-BM	Not tested	20 μM	Pre-clinical	[219]
	Phorbaketal A	Stimulation of osteoblastogenesis via TAZ-mediated RUNX2 activation	Porifera	C3H10T1/2	Not tested	1 μg/mL	Pre-clinical	[220]
	Phorbasone A,B	Stimulation of ECM mineralization and osteoblastogenesis. Upregulation of osteoblast markers *RUNX2, ALPL, SP7,* and* PTH/PTHLH*	Porifera	C3H10T1/2	Not tested	0.25 μg/mL	Pre-clinical	[221]
	Aerophobin-1	Increased mineralization of vertebral bodies in zebrafish larvae	Porifera	Zebrafish embryo	Not tested	0.1 μM (zebrafish)	Pre-clinical	[222]
	Norzoanthamine (and truncated form)	Protective effect against collagen I, elastin and BSA degradation. Acceleration of hydroxyapatite crystals formation. Inhibition of nitric oxide production. Inflammation-suppressive effect through the inhibition of MAPK pathway and COX-2 and iNOS expression	Cnidaria	C3H10T1/2; MC3T3-E1	OVX rat; OVX mouse	Intramedullary PLGA-PEG implants 1 mg/3 weeks (rat) 2 mg/kg/day (mouse)	Pre-clinical	[223]
	7β-Hydroxy-8α-methoxy-deepoxysarcophytoxide	Stimulation of osteoblastogenesis, collagen content, ALP activity and nodules formation	Cnidaria	MC3T3-E1	Not tested	0.3 μM	Pre-clinical	[224]
	Sarcomilasterol	Stimulation of osteoblast proliferation and ECM mineralization. Upregulation of osteoblast marker ALP	Cnidaria	MC3T3-E1	Not tested	3 μM	Pre-clinical	[225]
	Blue mussel-derived octapeptides FSVVPSPK and PIISVYWK	Stimulation of osteoblastogenesis through the stimulation of Wnt/β-catenin pathway in human cells. Stimulation of osteoblastogenesis and ECM mineralization through the stimulation of MAPK and BMP pathways in mouse cells. Attenuated cortical bone loss and reduced bone resorption markers in OVX-mice	Mollusca	hMSC-BM; mMSC-BM	OVX mouse	50 μg/25 mg /day (mouse)	Pre-clinical	[226, 227]
	Oyster-derived octa-peptide YRGDVVPK	Stimulation of osteoblast proliferation and differentiation, and ECM mineralization	Mollusca	MC3T3-E1	Not tested	0.1 μM	Pre-clinical	[228]
	Compound pearl protein polypeptide (CPPP)	Stimulation of osteoblast proliferation and differentiation, and ECM mineralization in osteoblasts. Increased BMD and serum levels of E2 and TGF-β1, downregulation of bone resorption markers (serum ALP and BGLAP and urine calcium and phosphorus) in OVX rats	Mollusca	Rat calvaria primary osteoblasts	OVX rat	20 mg/kg/day (rat)	Pre-clinical	[229]
	*N*-acetyl-*D*-glucosamine (NAG, chitin-derived)	Stimulation of osteoblast proliferation and differentiation, and ECM mineralization. Protective effect against H_2_O_2_-induced oxidative damage. Reduction of OVX-induced weight gain and uterine coefficient. Increased serum levels of calcium and ALP. Improved BMD, bone mechanical properties, tibia microarchitecture and histological features in OVX rats	Arthropoda	MC3T3-E1	OVX rat	250 mg/kg/day (rat)	Clinical for other diseases Pre-clinical for OP	[230]
	Tripeptide Leu-Pro-Lys	Stimulation of osteoblast differentiation and ECM mineralization through estrogen/ MAPK pathway	Anellida	C3H10	Not tested	25 μg/mL	Pre-clinical	[231]
	*Stichopus japonicus* polysaccharide SP-2	Increased osteoblastogenesis and ECM mineralization though the activation of BMP pathway	Echinodermata	MC3T3-E1	Not tested	1 μg/mL	Pre-clinical	[232]
	Pardaxin	Upregulation of BMP-2 and downstream markers of osteoblastogenesis (*Runx2*, *Sp7*, *Bglap*, Akt and ERK phosphorylation, ALP activity). Increased ECM mineralization in vitro. Increased number of mineralized vertebral bodies and increased mineralization of cranial skeletal structures. Upregulation of RUNX2, MMP-2, SP7 in GIOP zebrafish larvae	Chordata (Fish)	MC3T3-E1	Zebrafish model of GIOP	0.005 μM waterborne (zebrafish)	Pre-clinical	[233]
	Tripeptide Lys‐Ser‐Ala	Stimulation of osteoblast proliferation and differentiation, and ECM mineralization. Upregulation of osteoblast markers (BGLAP, SPP1). Stimulation of MAPK and Smad pathways via binding to BMP-2 receptors	Chordata (Fish)	MC3T3-E1	Not tested	400 μM	Pre-clinical	[234]
	Glycosaminoglycans rich in chondroitin and dermatan sulfates (H-CS/DS-GAGs)	Stimulation of osteoblast differentiation and ECM mineralization	Chordata (Fish)	MC3T3-E1; Calvaria primary osteoblasts	Not tested	25 μg/well (12-well plate)	Pre-clinical	[235]
	*Ciona intestinalis* calcitonin-like peptide	Stimulation of osteoblast proliferation and differentiation, and ECM mineralization though the activation of MAPK pathway	Chordata	MC3T3-E1	Not tested	7.5 μM	Pre-clinical	[236]
Dual-action	Macrolactin F	Inhibition of RANKL-induced osteoclastogenesis, F-actin ring formation, resorption activity, and downregulation of Akt, JNK and p38 pathways and osteoclast markers in macrophages. Promotion of nodule formation, upregulation of osteoblast markers and ALP activity, and activation of Akt and Smad pathways in osteoblasts	Actinobacteria	mDM-BM; MC3T3-E1	Not tested	10 μM (dual action)	Pre-clinical	[237]
	Macrolactin A	Inhibition of RANKL-induced osteoclastogenesis, bone resorption and actin ring formation. Downregulation of osteoclast markers (CTSK, ACP5, *Mmp2*, MMP9, NFATC1, FOS) through the inhibition of MAPK/Akt pathways in macrophages. Stimulation of osteoblastogenesis and ECM mineralization. Upregulation of osteoblast markers (ALP activity, *RUNX2, BMP-2, SP7, SMAD4, SPP1* expression) though the stimulation of MAPK/Akt pathways in osteoblasts. Improved BMD, bone volume, bone histopathological features, and increased TRAP + cells in LPS-induced osteoporotic mice	Actinobacteria	mDM-BM; MC3T3-E1	Mouse model of LPS-induced bone loss	50 mg/kg/day (mouse)	Pre-clinical	[238]
	Fucoidan	Stimulation of osteoblastogenesis and mineralized nodule formation; Upregulation of osteoblast markers (*Alp* levels and ALP activity; BMP-2 and BGLAP expression). Suppression of RANKL-induced osteoclastogenesis; Reduced number of nuclei per osteoclast, bone resorption and downregulation of osteoclast markers (*Acp5*, *Nfatc1*, *Oscar*, *Mmp9*)	Phaeophyceae (Heterokonta)	MG-63; RAW 264.7	OVX rat	5 mg/kg/day (rat)	Clinical for other diseases Pre-clinical for OP	[239, 240]
	Fucosterol	Increased osteoblast proliferation, ECM mineralization and ALP activity. Reduced RANKL-induced osteoclastogenesis. Downregulation of RANK receptor. Improved BMD and bone microarchitecture, increased serum levels of osteocalcin and decreased serum levels of CTX in OVX rats	Phaeophyceae (Heterokonta)	MG-63; mDM-BM	OVX rat	25 mg/kg/day (rat)	Pre-clinical	[241]
	Diphlorethohydroxy-carmalol	Suppression of RANKL-induced osteoclastogenesis via the inhibition of NF-κB pathway in macrophages. Protection against H_2_O_2_-induced oxidative cell toxicity and ROS generation. Increased ALP activity and nodules formation. Upregulation of *Col1a1, Alpl, Smad1/5, Sp7, Bmp2, Runx2* in osteoblasts	Phaeophyceae (Heterokonta)	mDM-BM; MC3T3-E1	Not tested	25 mg/mL (anti-osteoclatogenic) 0.2 mM (antioxidant)	Pre-clinical	[242, 243]
	Alginate oligosaccharide	Stimulation of osteoblast proliferation. Increased serum level of PTH1-84 and VEGF. Increased BMD in OVX rats. Increased BMD and enlarged trabeculae in D-galactose induced osteoporotic mice. Downregulation of senescence biomarker p53 expression. Inhibition of RANKL/RANK/C-Fos pathway and reduced NF-κB nuclear translocation. Reduced serum levels of osteocalcin. Upregulation of osteoprotegerin expression	Phaeophyceae (Heterokonta)	MG-63	OVX rat; Mouse model of D-galactose-induced bone loss	5 mg/kg/day (rat)	Clinical for cystic fibrosis Pre-clinical for OP	[244]
	Ishophloroglucin A	Increased osteoblast differentiation through the stimulation of MAPK pathway. Inhibition of osteoclastic differentiation through the inhibition of ERK and NF-κB pathways	Phaeophyceae (Heterokonta)	MG-63; RAW 264.7	Not tested	5 μg/mL (anti-osteoclastogenic) 6.25 μg/mL (pro-osteoblastogenic)	Pre-clinical	[245]
	Astaxanthin	Inhibition of RANKL-induced osteoclastogenesis via the downregulation of *Nfatc1*, *Acp*5, *Ctsk*, *Dcstamp* expression in macrophages. Increased osteoblastogenesis through AhR pathway and upregulation of *CYP1A1*, *BGLAP*, *SPP1*, *COL1A1*, *RUNX2* expression in MG-63. Stimulation of osteoblastogenesis and ECM mineralization through fatty acid metabolism regulation in MSC-BMs. Reduced serum levels of bone resorption markers. Increased BMD and microarchitecture of trabecular bone in OVX mice	Chlorophyceae (Chlorophyta)	mDM-BM; MG-63; rMSC-BM	OVX mouse	10 mg/kg/day (mouse)	Clinical for OA and joint inflammation	[246, 247]
	Austalide K	Reduction of RANKL-induced osteoclastogenesis in macrophages through the inhibition of NFATc1 expression at protein and gene level. Stimulation of BMP-2-induced osteoblastogenesis in myoblasts through the downregulation of *Runx2*, *Bglap*, *Spp1* expression	Eurotiomycetes (Ascomycota)	mDM-BM; C2C12	Mouse model of LPS-induced inflammatory osteolysis	2 mg/kg/day (mouse)	Pre-clinical	[248]
	Hymenialdisine	Reduction of RANKL-induced osteoclastogenesis, bone resorption and osteoclast differentiation through the inhibition of NF-κB and MAPK pathways, and NFATc1 expression. Stimulation of osteoblastogenesis and ECM mineralization through the activation of GSK-3β/β-catenin/TCF/LEF pathway. Upregulation of *Runx-2*, *Col1a1* and *Bglap*, and ALP activity	Porifera	mDM-BM; RAW 264.7; MC3T3-E1	OVX-Mice (phenotype prevented)	2 mg/kg/day (mouse)	Pre-clinical	[249]
	Blue mussel-derived dodecapeptide (IEELEEELEAER)	Stimulation of osteoblast proliferation and differentiation. Inhibition of RANKL-induced osteoclastogenesis. Prevention of cortical bone loss in OVX mice	Mollusca	MC3T3-E1; RAW 264.7	OVX mouse	30 mg/kg/day (mouse)	Pre-clinical	[250, 251]
	Compound amino acid-chelated calcium (CAA-Ca)	Increased BMD and bone calcium content. Improved bone architecture. Reduced serum and levels of bone resorption markers. Stimulation of Wnt pathway	Mollusca	–	OVX rat	100 mg/kg/day (rat)	Pre-clinical	[252]
Anti-osteonecrotic	Polydeoxyribonucleotide	Resolved osteonecrosis. Increased bone vascularization, osteoclast population, bone remodeling and bone volume. Improved bone microarchitecture	Chordata (Fish)	–	Rat model of bisphosphonate-related osteone-crosis of the jaw	1 mg/kg/day (rat)	Clinical for scars, ulcers, and sclerotic diseases	[253]

^a^RAW 264.7, mouse macrophage cell line, * MC3T3-E1* mouse pre-osteoblast cell line;* C2C12* mouse myoblast cell line;* MG-63* human osteosarcoma cell line;* C3H10* mouse embryo cell line;* C3H10T1/2* mouse embryonic fibroblast cell line;* 3T3-L1* mouse fibroblasts cell line;* D1* mouse bone marrow mesenchymal cell line;* mDM-BM* murine bone marrow-derived macrophages;* MSC-BM* bone marrow mesenchymal stroma/stem cells from mouse (*mMSC-BM*), human (*hMSC-BM*) or rat (*rMSC-BM*);* BMD* bone mineral density;* OA* osteoarthritis;* ROS* reactive oxygen species;* ECM* extracellular matrix;* OVX* ovariectomized

^b^Bioactive concentrations refer to in vivo data when available

Interestingly, MOC distribution resembles the tendency previously described for all-type marine bioactives [161], an indication that a similar sampling effort was directed toward these groups. Also of interest, ten of the fungi-related MOCs were isolated from species that live in close symbiotic relationships with marine sponges (5), corals (3), seaweeds (1), and mangroves (1).

## Future perspectives

### Underexplored groups as promising sources of MOCs

Many groups of marine organisms are underrepresented in the current screening scenario. Among those, marine algae have provided a plethora of bioactive compounds [254], and several studies support the idea that they represent a promising source of pharmacologically relevant osteoactive compounds. In this regard, mineral-rich extracts prepared from the red coralline algae *Lithothamnion* spp. have pro-mineralogenic properties that partly rescue bone loss in osteoporotic animal models [255]. Extracts prepared from green (*Codium fragile* and *Cladophora rupestris*) [303 and red (*Plocamium cartilagineum* and *Ceramium secundatum*) [256] macroalgae also showed pro-mineralogenic activity in fish osteochondroprogenitor cells and pro-osteogenic activity in zebrafish. Red (*Dichotomaria obtusata*) and brown (*Padina pavonica*) macroalgae triggered pro-osteoblastogenic signals in mouse bone marrow MSCs [257] and human primary osteoblasts [258]. Recently, calcium-chelating peptides derived from several species of marine microalgae could rescue osteoporotic phenotypes in zebrafish [259]. It is worth mentioning that the large-scale production of algal biomass is supported by a well-established and technologically advanced industry. Of special interest, microalgae have been long cultivated for nutritional, biotechnological, and industrial applications and are being used for the production of food, dietary supplements, cosmetics, pharmaceuticals, biofuel, fertilizers, but also for wastewater treatment [260]. Following important biotechnological advancements that improved growth conditions and allowed the establishment of genetically modified strains optimized for growth and compound biosynthesis [261], microalgae are expected to become highly relevant species for marine pharmacology in the upcoming years. In this regard, ethanolic extracts prepared from two species of microalgae (*Skeletonema costatum* and *Tetraselmis striata*) were recently shown to contain potent osteoactive compounds [262].

Marine invertebrates such as mollusks, gastropods, and echinoderms are also promising sources of osteoactive compounds. Among the mollusks, bivalves such as mussels, oysters, clams, and scallops have originated peptides, polysaccharides, and glycoproteins with antioxidant and anti-inflammatory activity, and lipids and polyunsaturated fatty acids with strong anti-inflammatory and anti-arthritic properties [263]. Osteoanabolic [250] and antiresorptive compounds have also been isolated from bivalves. Among those, the nacre, also known as mother of pearl, has both osteoinductive and antiresorptive properties [264, 265]. Fermented extracts of the oyster *Crassostrea gigas* have also a dual-action activity, stimulating osteogenic differentiation via Wnt and IGF pathways [266, 267] and suppressing osteoclast differentiation, thus preventing OVX-induced bone loss in mouse [268]. Similarly, aqueous extracts of the bivalve *Pisidium coreanum* showed anti-osteoclastogenic activity and were able to rescue osteoporosis in OVX mice [269]. Among the gastropods, methanolic extracts of the brown dwarf turban (*Turbo brunneus*) and the sea snail *Euchelus asper* prevented bone loss [270] and improved osteoporotic phenotype [271], respectively, in OVX mice. Echinoderms such as sea urchins, starfish and sea cucumbers are at the origin of about 5% of all the marine bioactives discovered so far [161]. In the context of this review, polyhydroxylated naphthoquinones extracted from the sea urchin *Evechinus chloroticus* increased ECM mineralization in human osteosarcoma cells when administered together with calcium chloride, but decreased it when administered alone [272]. Sea cucumbers also hold a great deal of potential with both osteoanabolic [273] and antiresorptive [274] extracts identified.

Among chordates, ascidians such as sea squirts are well-known sources of compounds with anticancer, antimicrobial, and antioxidant activities, some of which are being currently evaluated in clinical trials [275]. Compounds with osteoactive properties have also been isolated from ascidians [199, 236], and extracts with antioxidant and anti-inflammatory activities have recently been found to also exhibit pro-osteogenic properties [276]. In vertebrates, bone-derived gelatin from the saffron cod (*Eleginus gracilis*) and skin-derived gelatin from the blue shark (*Prionace glauca*) have shown protective properties against bone loss in OVX rats [277, 278], while bone powder from tuna (*Thunnus* spp.*)* could reduce bone loss in a GIOP mice through the co-regulation of NF-κB and Wnt/β-catenin pathways and the modulation of gut microbiota composition and metabolism [279].

Finally, dichloromethane and ethanolic extracts of halophyte plants *Salicornia herbacea* and *Spergularia marina*, respectively, were reported to have anti-adipogenic and pro-osteoblastogenic activities in vitro [280, 281]. Recently, polyphenols-rich extracts of *Spartina alterniflora* and *Salicornia fragilis* were found to have pro-mineralogenic activity in fish osteochondroprogenitor cells and pro-osteogenic activity in zebrafish [282].

### The availability of animal models and screening tools is not fully exploited

**Fig. 4:**
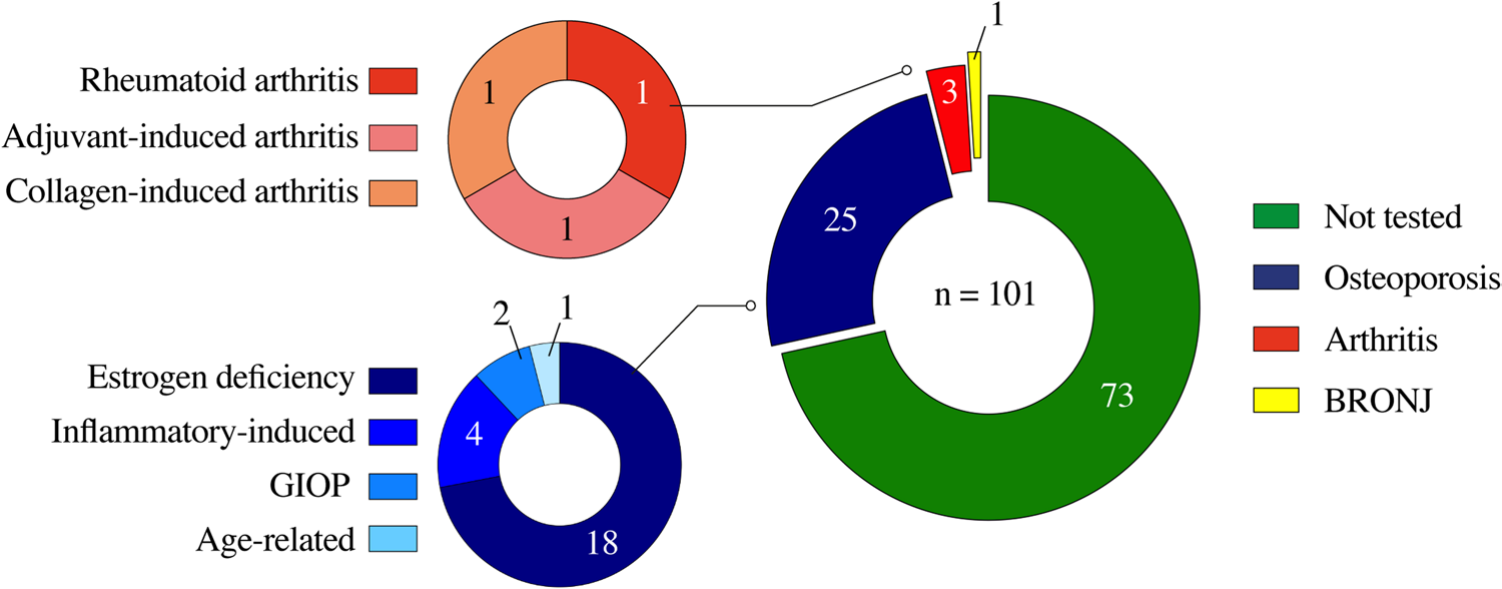
Distribution of the marine osteoactive compounds (MOCs) based on the animal disease model used for validation. BRONJ, bisphosphonate-related osteonecrosis of the jaw; GIOP, glucocorticoid-induced osteoporosis

The global interest for underexplored marine organisms as a source of osteoactive compounds has steadily increased in the last 2 decades following the demonstration that they produce osteoanabolic and antiresorptive compounds. However, the discovery of novel MOCs is only achievable through a coordinated effort that should aim at the fractionation of the extracts, isolation, and identification of the osteoactive compounds, together with the validation of their biological activity and the elucidation of their mechanisms of action. In this aspect, animal models are increasingly available for compound validation, although only 28% of the compounds listed here were validated in an animal model of metabolic bone disorders (Fig. 4), while the vast majority, i.e., 72%, were only tested in vitro, mainly using rodent cell lines. Of the compounds that were validated using in vivo disease models, 25 were tested in animal models of osteoporosis, 3 were tested in mouse models of arthritis, and 1 was tested in a model of bisphosphonate-related osteonecrosis of the jaw. None were tested in animal models of VD-deficiency, hyperparathyroidism, Paget’s disease of bone, or osteopetrosis. Of the compounds tested in animal models of osteoporosis, 18 were tested in rodent models of ovariectomy-induced osteoporosis, 4 were tested in mouse models of LPS-induced bone loss, 1 in a mouse model of D-galactose-induced osteoporosis, and 2 in a zebrafish model of glucocorticoid-induced osteoporosis. In this context, rodents and in particular the mouse, are the preferred animal models in biomedical research due to their genetic similarity with humans, small size, short lifespan, and relatively low maintenance cost compared to other mammalian models [283]. A large variety of mouse models mimicking skeletal disorders are available. The ovariectomized rat and mouse, aim at resembling mechanistically the pathophysiology of postmenopausal osteoporosis and are considered gold-standard in vivo models to validate the efficacy of compounds and drugs with anti-osteoporotic potential [284]. Mouse models that resemble age-related osteoporosis [285], male senile osteoporosis [286], and GIOP [287] are also available to researchers but none of these models have yet been implemented to evaluate the efficacy of MOCs. A rat model of bisphosphonate-related osteonecrosis of the jaw [288] has been successfully used to validate the anti-necrotic potential of a salmon sperm-derived polydeoxyribonucleotide [253]. Great achievements have also been obtained in the modeling of disorders of mineral homeostasis, including vitamin D deficiency [289], primary hyperparathyroidism [290], and renal osteodystrophy [291] using rodents. Models have also been developed for bone genetic disorders such as PDB [292] and osteopetrosis [293].

However, rodent models have technical disadvantages that limit the throughput of screening pipelines for drug discovery. When compared to fish and invertebrate models, rodent systems bring the complexity and the genetic proximity that better resemble humans but are also expensive and more time-consuming. As such, they may be better suited for secondary screenings that aim at validating compound osteoactivity, rather than for primary screenings that mostly serve at funneling down the number of compounds. Teleost fish, in particular the zebrafish (*Danio rerio*) and the Japanese medaka (*Oryzias latipes*), are becoming extremely relevant in bone research and can model many human skeletal diseases [294, 295]. These small teleosts offer several technical advantages that make them well suited for drug screening, e.g., smaller size, cost-effectiveness, shorter life span, and higher fecundity when compared to mammalian models. Moreover, the translucency of embryonic stages throughout development and the amenability to gene editing has enabled the generation of a vast array of transgenic and mutant lines that can be used for in vivo-cell tracking and disease modeling [296]. Furthermore, teleost ability to regenerate bone and cartilage tissues offer a different approach for evaluating the osteoactivity of drugs and compounds [297]. As such, a large numbers of drug screening tools have been developed in the latest years based on teleost fish [298, 298], offering a cost-effective, medium- and high-throughput alternative to mammalian-based systems and at the same time providing a level of biological complexity which cannot be yet achieved by in vitro systems. Importantly, several zebrafish and medaka models of human bone disorders are available, including osteoporosis [299], osteopetrosis [300], and PDB [301]. However, teleost models such as zebrafish pose various challenges, including the higher evolutionary distance with humans compared to classical mammalian models, that oftentimes reflects into physiological and anatomical differences [302]. Though, the great advantages offered by these animal models make them very efficient intermediate points between exploratory screening and functional validation of novel osteoactive compounds. Owning to this variety of animal models, it is expected that, in the coming years, the research community working in the field of marine osteoactive compounds will fill the gap in terms of in vivo validation of MOCs.

## Conclusion

Metabolic bone disorders and fragility fractures are major causes of reduced welfare, suffering, and morbidity, as well as a tremendous sink of resources for the global health systems. Because most of the drugs currently available are associated with undesirable side effects, there is an unmet demand for effective medications to address metabolic bone disorders. Oceans are increasingly contributing to pharmaceutical research and drug discovery and may hold the solutions to resolve this pressing issue through the production of novel and innovative osteoactive compounds by marine organisms. Our survey of the literature on marine osteoactive compounds identified 101 compounds with antiresorptive, osteoanabolic, or anti-osteonecrotic activities, including compounds with dual activity. It also revealed that marine invertebrates, such as sponges and cnidarians, and microorganisms, such as fungi and cyanobacteria, are major contributors of MOCs, and that future research efforts should explore the untapped biodiversity of marine organisms, such as microalgae, mollusks, holothurians, ascidians, and fishes. To achieve these goals, a cooperative effort between the chemical characterization of marine-derived compounds and the exploitation of drug screening and validation tools currently available will be necessary.

## Data Availability

All datasets generated and/or analyzed during the current study are available from the corresponding author on reasonable request.
